# Observations on Curative Evidence

**Published:** 1856-01

**Authors:** J. R. Black

**Affiliations:** Linnville, O.


*Observations on Curative Evidence.*—By J. R. Black, M. D., Linnville, O.

Mr. Editor:—There are but few men, with a sprinkling of gray hairs, that have not a remedy of undoubted efficacy, for some specific disease. They will tell, with a self-satisfied air, of the case or cases in which the medicine operated, and the peculiar certain manner in which it subdued the disorder. It is difficult for the physician to repress a smile at the credulity of his informant, and as for his hinting that they may be mistaken, it only brings out new asseverations of what they claim to be perfectly certain of; for what they know, they know—and that is the alpha and omega of the matter. The evidence on which such cures rest, does not convince the physician who is well aware where the fallacy lies in such cases. Yet, it is well to inquire if the credulity of the public does not also extend to the profession in regard to the curative virtues of numerous articles. That this is probable, may be inferred from the fact, that in the art of medicine, the difficulties of arriving at the strict truth, are unparalleledly great. Even the most astute minds are free to acknowledge how little is really known; how liable they are to error amid the mass of causes and counter-causes, of effects and counter-effects, in which they have to labor, and if the veil be thus frequently drawn to penetrating genius, how much oftener must the ordinary practitioner be groping in the dark? This, however, will only serve to stimulate the diligent physician to render his art more luminous and perfect. As an example of easy credulity, take the following: A self-made doctor was called, several years ago, to see a child laboring under sudden nervous and gastric disturbance. The child had been seen with some tomatoes in its hand, which it had, in all probability, ate. He immediately pronounced the case (agreeable to the notions of that day) one of poisoning from that delicious esculent, and addressed his remedies accordingly, until the medicine, as he said, destroyed the poison, and then, rubbing his hands with satisfaction, pronounced the case *cured.*

It is often a point of infinite difficulty with the conscientious physician, precisely to distinguish between the relative claims of his medicine and that of *vis medicatrix naturæ*, in curing a disease. To follow a routine of business, and complacently conclude that the majority, if not all, the symptoms of amendment are due to the medicines employed, would be an empyrical, easy and reprehensible way of dealing with the physician's avocation. A prominent exemplification of routine practice is seen in those who give mercurials in every case in which it is possibly admissible, the question not being what medicine is best adapted to the case, but *can* mercurials be given. Although the success from this course may be shown to be good, yet it has not been proved to be the best. Nay, more; it is a practice fraught with obloquy to the profession, and we think will soon be, from the light of accumulating evidence, among the things that were. Some of this evidence, it is presumable, is within every one's reach. One physician will place his whole reliance on calomel, in quite a variety of diseases, while another will eschew it entirely: both are probably wrong, yet so far as the writer's observations extend, the modicum of success is with the latter. Be it said, however, that by the latter is not meant those vaunting empyrics who magnify themselves by the magnitude of those they assail, but men that claim only the name of physician, and who would scorn to ride into notoriety on the *woolly* hobby horses of the "isms" and "pathys."

If evidence were closely sifted, there would not, nor could there be such radical differences as to treatment. Such a rigid inquiry, however, is not consonant with the feelings of many followers of the art. They have obtained a good reputation for skill, and are built up by their popularity. They care not, therefore, to adjudge their case different from the prevailing sentiment. This class of physicians are the very ones liable to fall into a fixed routine of practice, without much regard to the cycle of diseases, the epidemic influence, or, in fact, to many fundamental pathological principles. It is a misfortune with our profession that, if a man once gets his name up for skill, it will only be by the most uncommon circumstance that it will ever be brought down. The proverb of "get a name for early rising, and you may lie abed all day," was never truer than when applied to the name a physician once gets. Hence it is, that those not noted for purity of actuating motives, will continue the same line of remedies for scores of years together, almost regardless of any advances made by his professional brethren. Under the gloss of a name, such will often heap disgrace on science, and give room to countenance those quackish patantees, whose ignorance is only equalled by their audacity. The writer is aware of numerous cases of a chronic nature, in which some M. D. seemed as if he could not give them medicine unless it was a mercurial preparation, or some one of the potent antiphlogistic list—even after the patients had already been bled, blistered, purged, puked and salivated. This course only seemed to frustrate nature's efforts, the final result being, that one of the "pathys" would be seen, and he, with a general anathema on "mineral pisin doctors," would administer some inert substance, equivalent to nothing, and thus give fair opportunity to the *vis medicatrix naturæ.* In this manner, the quack gets the credit of cure; nature gets none; the "pathy" gains a friend, the M. D. an enemy.

The seeker for truth will not soon forget the occasional, but decided lessons of nature in the treatment of disease; one such transpired to the writer not long since. Being called to see a man, of medium constitutional strength, and learning from the friends that he had labored for three weeks under a fever, the type of which they knew not, and that now he was down from a relapse of what appeared to be a favorable convalescence, I found him presenting the following symptoms: Skin dry, and of a pungent heat—pulse 126—breathing nearly normal: patient lying on his back with a stupid delirium—at times great restlessness and tossing of the arms—tongue dry, and covered in the centre with a dark crust—bowels has not moved for thirty-six hours. Ordered a purge of infusion senna with sulphate magnesia, to be followed by the neutral mixture every two hours; cold water to be applied freely to the head. Twenty-four hours after, saw him again; medicine operated freely, bringing away dark dejection; symptoms otherwise materially unchanged; continued neutral mixture. Thirty hours later found no amendment; delirium increased, and the tongue drier, with indications of a general failure of the secretions. Sub. murias and ipecac. āā three grs. every four hours. Twenty-six hours after, found the most marked amendment: skin moist and cold; tongue cleaning; pulse 100; delirium gone, and had asked for nourishment. Congratulating the friends on the progress of the case, and remarking the excellent effect the last medicine had on the disease, when, to my astonishment and confusion, learned that they had not been able to get a particle of the last prescription down him. What, not a grain? No, not one; he clenched his teeth, and resisted so much, that trying was desisted from, for which the patient and friends expressed due penitence.

Attentively considering such evidence, it is impossible to resist the conclusion (*a*) that to nature is often due the *primum mobile* of curative action, and (*b*) that in certain cases the physician's remedies are in advance of the curative action by nature.

(*a*) In the greater number of zymotic diseases, the most opposite systems of medication have not, so far as the writer is aware, produced a corresponding contrast in final results. From local knowledge, and the reports found in the medical journals, it appears that in the treatment of dysentery, for instance, the widest imaginable range has been taken in the selection of remedies. One treats it with acids, another with alkalies; one with mercury and opium, another with pure astringents, while yet another will use castor oil or turpentine, or, as the writer has known, rely on a judicious selection of aromatics. Each dispenser of his remedy thinks his own remedy the best, although it is seldom that any one of them can show that any special course has indisputable advantages over all others. Again: in the treatment of cholera, what an array of medicines, embracing some of every class, do medical journals show; yet it is, at this moment, impossible to point out *the* remedy that can best claim that appellation. There are other examples that might be given to sustain the opinion that the *vis medicatrix naturæ* is the prime moving force in remedying certain diseases, that the physician's appliances are not remedies, but mere correctives or accessories to nature's efforts, and in addition, that it is folly to seek for a specific in these cases; the physician's duty being limited to discover what aids he can select that may best assist nature without detriment to the vital powers.

(*b*) But there is a class of diseases in which the physician's appliances are remedies; or, in other words, diseases are cured for which the natural powers are inadequate, and in which the vital powers play a subordinate part. As instances of this class, scurvey, syphilis and scrofula may be referred to, although there are many other disorders unequivocally under the control of medicine, but these are sufficient to place in contrast with those in which nature is the main physician. The treatment of these diseases differs but little throughout the civilized world, presenting nearly an entire unanimity as to the best mode of procedure. The evidence here on which cures rest, is excellent. There are no exacerbations of vital or morbid movements—the disease running one steady course, generally impassive, until *the* article is administered that produces an immediate amendment. It is not intended, here, to carry the distinction further, nor to give any approach to an accurate list of the diseases that properly belong to each class, but simply to call attention to the distinction. Perhaps, were it kept more prominently in view, together with the inferences that may legitimately be drawn therefrom, there might be less cause to complain of the quackery so rife in the land, and the equality of respect the public so often alike accord to the scientific physician and the most noted charlatan. Perhaps fewer physicians would strive to follow the popular idea of cutting short certain diseases by the use of the most powerful remedies. There might be fewer examples of that marasmus of constitution so common after severe ptyalism. The empiric would seldom acquire that reputation for skill that he does from the marked amendment many patients show as they get from the severe and potent treatment of the regular, to the mild and simple treatment of the quack.

The credit of the Homeopathist is mainly due to the blind adherence of the instructed physician in nearly every case to the severe and heroic treatment. Nature is kept down, and overpowered by the most active remedies of the materia medica, until that infinitessimal creature of Hahnemann gets charge of the case, and he, by knowing nothing, and by doing the next thing to it, gives nature unrestricted play, inducing, as a natural consequence, an improved operation of vital power, for which Mr. Sugar Pills gets immense credit. The patient, of course, refers all the improvement to the magic pills, whereas the truth is, that the mere withdrawal of the most *acute* remedies in treating a *chronic* case, produces the change. If a candid empiric, taking it for granted that there *may* be one, were questioned as to the cases that gained him the greatest notoriety, the answer would doubtless be, that they were those that had been thus treated by the regular physician. The reality of cures from very minute doses often apparently depends upon their administration. Indeed, the majority of the public who generally refer all improvements of a disordered system to some tangible agent, are fit subjects to convert the appearance into a reality. It is only the intelligent and experienced physician that is capable of appreciating the truth in such cases, and we trust that they will act more upon the knowledge.—*Med. Counsellor.*

